# Concomitant brain and liver abscesses: a rare complication of acute diverticulitis

**DOI:** 10.1093/jscr/rjac297

**Published:** 2022-06-28

**Authors:** Izziddine Vial, Timothy Varghese, Adnan Sheikh

**Affiliations:** Department of General Surgery, East Lancashire NHS Trust, Blackburn, UK; Department of General Surgery, East Lancashire NHS Trust, Blackburn, UK; Department of General Surgery, East Lancashire NHS Trust, Blackburn, UK

**Keywords:** diverticulitis, abscess, bacteraemia, brain, liver

## Abstract

Diverticular disease is one of the most common colonic pathologies in the Western world. In the UK, ~80% of the population aged over 85 years are diagnosed with it. Most of these cases are asymptomatic. Yet, they can become problematic when the diverticula bleed, become infected (diverticulitis) or perforate. Other well-known complications of diverticular disease are acute inflammation, stenosis, fistulation and abscess formation. In this case report, we describe a delayed presentation of metastatic abscesses (liver and brain) from a prior acute diverticulitis with contained perforation and abscess formation.

## INTRODUCTION

Diverticulosis is a common colonic condition that is diagnosed in 50% of the US population over the age of 50 and in roughly 80% of people aged over 85 years in the UK [1–4]. Acute inflammation, stenosis, fistulation and abscess formation are common colonic complications of diverticular disease while extra-colonic pyogenic abscess formation in the liver or brain is far fewer. There are a handful of case reports in literature with synchronously concomitant liver and brain abscesses. This report aims to describe the atypical presentation of a complicating diverticulitis case.

## INITIAL PRESENTATION

### History and examination

A 55-year-old gentleman presented to the emergency department with a 2-week history of severe lower abdominal pain and distention. On examination, there was suprapubic distention, tenderness and localized peritonism in the lower abdomen. Initial blood results were as follows: white blood cells (WCC) 24.2 × 10^9^/l, neutrophil count (Neuts) 21.7 × 10^9^/l, lymphocytes (Lymph) 1.2 × 10^9^/l, C-reactive protein (CRP) 153 mg/l, alanine aminotransferase (ALT) 78 IU/L and platelets (Plts) 486 × 10^9^/l. Computer tomography (CT) revealed acute diverticulitis with a contained perforation and collection. A decision to carry out an emergency laparotomy and Hartmann’s procedure was made.

### Operation

A laparotomy was performed; intraoperatively surgeons found an acute sigmoid diverticulitis with abscess formation extending to the lower pelvis. There were multiple areas of small bowel adherent to the abscess cavity and the diverticular phlegmon; the adherent small bowel was not viable. This was resected and reconstructed with a side-to-side anastomosis. Sigmoid resection and end colostomy in the left iliac fossa were formed as routine.

### Postoperative course

Immediately postoperatively the patient was sent to the critical care unit. He made a good recovery and was discharged 1 week later. At his follow-up 4 weeks later, he complained of generalized malaise and significant weight loss. There were no abdominal signs of ongoing infection or other neurological symptoms to note.

**Figure 1:**
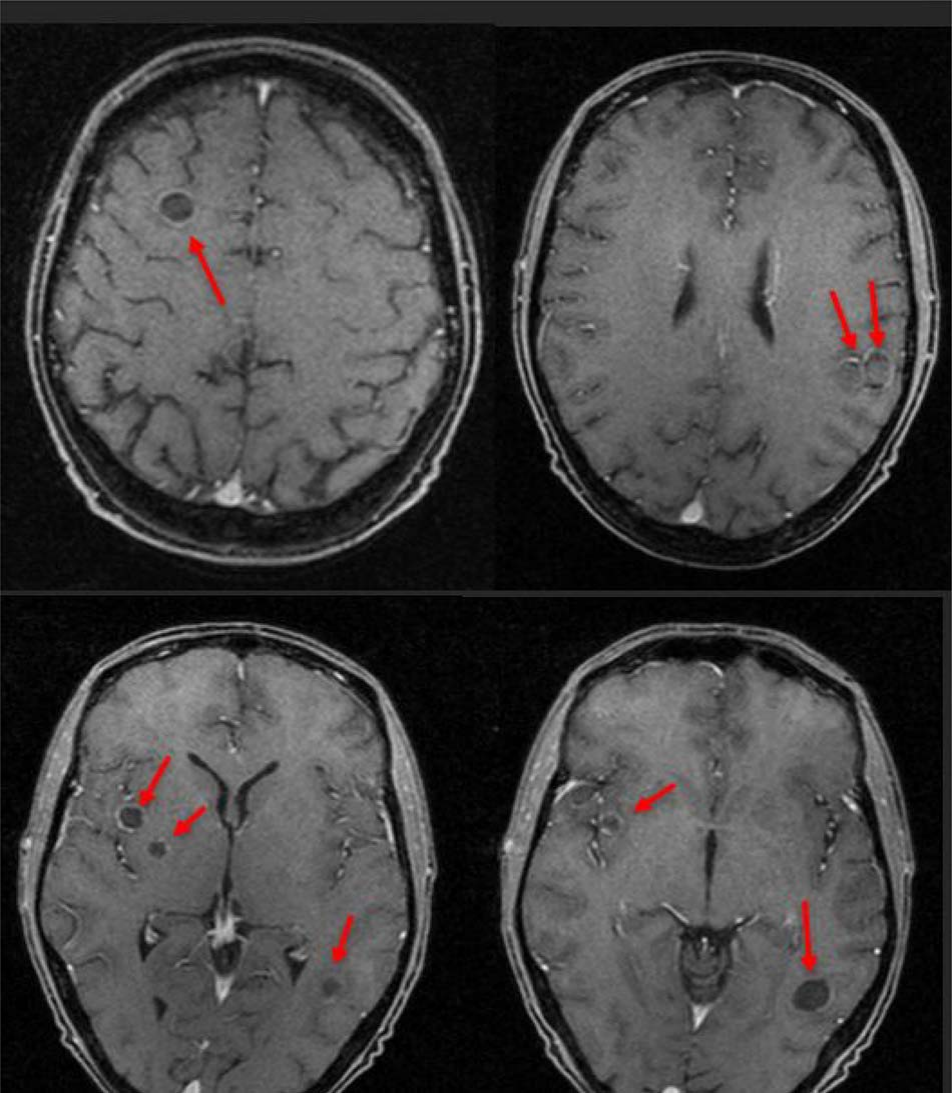
MRI scan after first seizure showing multiple brain abscesses.

**Figure 2:**
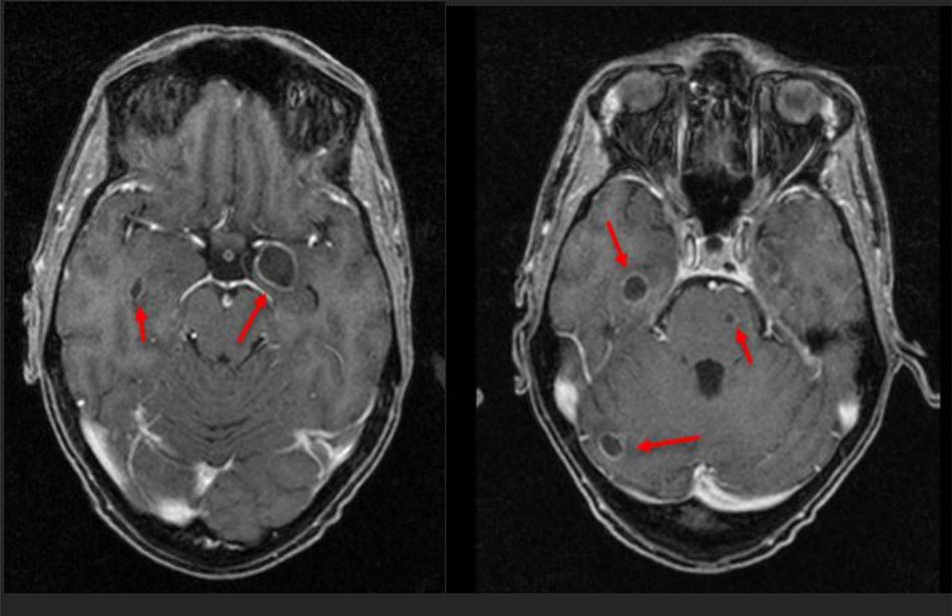
First MRI scan showing deeper brain abscesses.

Six days following his initial follow-up, this patient was brought to the emergency department by ambulance following a witnessed seizure. He was distressed and confused on arrival but otherwise apyrexial with a non-tender abdomen. Initial blood tests were as follows: haemoglobin 82 g/l, WCC 25.3 × 10^9^/l, CRP 284 mg/l, Plts 477 × 10^9^/l, Neuts 23.8 × 10^9^/l, Lymph 0.6 × 10^9^/l, bilirubin 30 μmol/l, albumin 266 g/l, ALT 133 IU/l and alkaline phosphatase 914 IU. All other blood tests were non-contributory. Blood cultures isolated a *Streptococcus intermedius* bacteraemia. CT revealed four large hepatic abscesses with the largest in segment 4b measuring 92 × 90 mm and a right hepatic vein thrombus. Three of the liver abscesses were drained to completeness by interventional radiology (IR). Magnetic resonance imaging (MRI) showed several focal restricting intraparenchymal brain lesions, posterior left temporal lobe measuring 1.7 cm and multiple smaller lesions within the right frontal, right para-ganglionic region, right parahippocampal, left uncal regions, right cerebellar hemisphere and left pons (Figs 1 and 2) consistent with brain abscesses.

**Table 1:** The literature regarding concomitant brain and liver abscesses from diverticulitis

Author and year	Age (years), sex	General surgery procedure	Either brain or liver abscess or both	Neurological symptoms (acute or subacute presentation)	Neurological symptoms—description	Organisms isolated	Abscess treatment
Dixon *et al*. 1989 [13]	64 Female	Not reported	Brain abscess only	Acute	Confused Fluent dysphasia Right hemiparesis Right upper motor neuron facial weakness	*Streptococcus milleri* group	Drainage (gentamicin + vancomycin inserted) + IV antibiotics treatment (Cefotaxime + gentamicin)
Valero *et al.* 2008 [11]	75 Male	Hartmann’s procedure	Brain abscess only	Acute	Fever Reduced consciousness	*Enterococcus faecalis*	Drainage + IV antibiotics (Cefotaxime + metronidazole + ampicillin)
Kamath *et al.* 2011 [5]	37 Male	Hartmann’s procedure	Liver, spine and multiple brain abscesses	Acute	Weakness of right lower limb—marked loss of power in the knee and ankle Sensory loss in the left leg	*Streptococcus intermedius*	Liver abscess—percutaneous drainage + IV antibiotics treatment (vancomycin + metronidazole + Imipenem + Clindamycin + Meropenem
Loyal *et al.* 2014 [10]	67 Female	Interventional radiological drainage + Hartmann’s procedure	Brain abscess only	Subacute	Confusion Garbled speech Right hemiparesis	Not reported	Stereotactic drainage + external ventricular drain
Al Mulla *et al.* 2020 [6]	55 Male	Not reported	Brain and liver abscesses	Acute	Persistent headache along with altered level of consciousness	*S. intermedius*	IV antibiotics

### Management

A multidisciplinary (MDT) decision was made to treat the remaining liver and brain abscesses conservatively with IV antibiotic therapy. The patient was commenced on anticoagulation for the hepatic vein thrombosis. Initial antibiotic therapy included amoxicillin, gentamicin and clindamycin for 7 days; this was reduced to an amoxicillin and metronidazole regime for seven more days. Amoxicillin was replaced by ceftriaxone for 14 days and linezolid was added for 28 days following a diagnosis of endocarditis. Metronidazole, ceftriaxone and linezolid resulted in a complete resolution of the liver abscess after 49 days of antibiotics.

An MRI head then showed that the intra-cerebral cavitating lesions were solidifying and reducing in size. There were also several new microabscesses present in the left temporoparietal region. A further 5-week intraveneous (IV) antibiotic therapy solidified the new lesions.

The patient was discharged with a further 4 weeks of an oral antibiotic therapy regime including co-trimoxazole and linezolid.

## DISCUSSION

Literature (Table 1) shows that cases of diverticulitis with concomitant brain and liver abscesses are extremely rare. Although the liver is a common site of abscess formation, second only to the pelvis in patients with diverticular disease, only two cases of concomitant brain and liver abscesses have been reported [5, 6, 7].

Brain abscesses usually arise secondary to penetrating trauma, otic infections, prior neurosurgical interventions or in the immunocompromised [8]. The incidence of brain abscess formation from extra-cerebral infection is reported at 0.4–0.9 per 100 000 persons [8] while paracolic abscess formation from diverticular disease has been reported to be around 7.4% in a 200 000 cohort study [9] with only five cases of brain abscesses arising from diverticular disease.

Loyal *et al*. reported the appearance of neurological symptoms 6 days (subacute) after sigmoidectomy [10]. These presentations are earlier relative to this case who presented nearly 6 weeks postoperatively with a seizure.

*Streptococcus intermedius*, *Streptococcus milleri* group and *Enterococcus faecalis* are usually found in the normal human gut. If they find their way in the bloodstream, they can become aggressive pathogens causing serious extraintestinal infections, which may not resolve if the original source of the infection is not removed. Intravenous antibiotics with surgical treatment by Hartmann’s procedure are indicated. This was done in three out of the five cases reported [5, 10, 11].

*Strep. intermedius*, subgroup of *Streptococcus milleri*, are frequently associated with abscess formation in the abdominal cavity and metastatic complications [12].

Doctors differed in their treatment plan for the brain abscesses. Some proceeded with neurosurgical or IR intervention while others opted for conservative management with IV antibiotics [5–8, 10, 11, 13].

## CONCLUSION

Diverticular complications can be diverse in their presentation. Though they more frequently present intra-abdominally, physicians should always be cautious about extra-abdominal complications too. There is still much heterogeneity in the management of extra abdominal complications and most management plans are case specific. It is important to consider the development of synchronously concomitant liver and brain in otherwise fit and well patients, as outlined in this report. Furthermore, clinicians may need to contemplate metastatic abscess formation early in the management of patients with severe diverticular disease.

## CONFLICT OF INTEREST STATEMENT

None declared.

## FUNDING

None.
