# Correction to: Concomitant brain and liver abscesses: a rare complication of acute diverticulitis

**DOI:** 10.1093/jscr/rjac556

**Published:** 2022-12-27

This is a correction to: Izziddine Vial, Timothy Varghese, Adnan Sheikh, Concomitant brain and liver abscesses: a rare complication of acute diverticulitis, *Journal of Surgical Case Reports*, Volume 2022, Issue 6, June 2022, rjac297, https://doi.org/10.1093/jscr/rjac297.

In the originally published version of this manuscript, there was no indication that Izziddine Vial and Timothy Varghese are joint first authors.

This error has now been corrected.

